# Genetic Variation in Attachment Glycoprotein Genes of Human Respiratory Syncytial Virus Subgroups A and B in Children in Recent Five Consecutive Years

**DOI:** 10.1371/journal.pone.0075020

**Published:** 2013-09-17

**Authors:** Guanglin Cui, Runan Zhu, Yuan Qian, Jie Deng, Linqing Zhao, Yu Sun, Fang Wang

**Affiliations:** 1 Laboratory of Virology, Peking University Capital Institute of Pediatrics Teaching Hospital, Beijing, China; 2 Laboratory of Virology, Capital Institute of Pediatrics, Beijing, China; University of Melbourne, Australia

## Abstract

Human respiratory syncytial virus (HRSV) outranks other viral agents as the cause of respiratory tract diseases in children worldwide. Molecular epidemiological study of the virus provides useful information for the development of globally effective vaccine. We investigated the circulating pattern and genetic variation in the attachment glycoprotein genes of HRSV in Beijing during 5 consecutive seasons from 2007 to 2012. Out of 19,942 tested specimens, 3,160 (15.8%) were HRSV antigen-positive. The incidence of HRSV infection in males was significantly higher than in females. Of the total 723 (23.1%) randomly selected HRSV antigen-positive samples, 462 (63.9%) and 239 (33.1%) samples were identified as subgroup A and B, respectively. Subgroups A and B co-circulated in the 5 consecutive HRSV seasons, which showed a shifting mixed pattern of subgroup dominance. Complete G gene sequences were obtained from 190 HRSV-A and 72 HRSV-B by PCR for phylogenetic analysis. Although 4 new genotypes, NA3 and NA4 for HRSV-A and BA-C and CB1 for HRSV-B, were identified here, they were not predominant; NA1 and BA9 were the prevailing HRSV-A and -B genotypes, respectively. We provide the first report of a 9 consecutive nucleotide insertion in 3 CB1 genotype strains. One Beijing strain of ON1 genotype with a 72 nucleotide insertion was found among samples collected in February 2012. The reversion of codon states in glycosylation sites to previous ones were found from HRSV strains in this study, suggesting an immune-escape strategy of this important virus.

## Introduction

Human respiratory syncytial virus (HRSV) is the most important viral agent of acute respiratory infections (ARI) in infants and young children [1] and vulnerable adults [2,3]. ARI is the leading killer of children under the age of 5 worldwide, especially in developing countries, causing 1.9 million deaths per year [4]. Recurrent infections with HRSV are common throughout life [5-7].

HRSV, a member of the *Pneumovirus* genus in *Paramyxoviridae* family of order *Mononegavirales*, consists of a non-segmented, single-stranded negative sense RNA genome packaged in a lipid envelope [8]. The genome of HRSV is approximately 15.2 kb and contains 10 genes encoding at least 11 proteins, among which the fusion (F) glycoprotein, the major attachment (G) protein and small hydrophobic (SH) protein are virus encoded transmembrane surface glycoproteins.

The G protein of HRSV is a type ii transmembrane glycoprotein containing cytoplasmic domain, transmembrane domain and ectodomian [9]. It varies in length from 282 to 321 amino acids [10-13]. The secreted form of G protein arises from the altered usage of translational start site [9,14]. The addition of O- and N- oligosaccharides to the G polypeptide backbone increases its molecular weight from 32 KDa to 80-90 KDa [15] and, most important, changes its antigenic characteristics [16,17]. The ectodomain of the G protein consists of two mucin-like highly variable regions, HVR1 and HVR2, separated by a 13 amino acid length domain that is highly conserved in almost all wild-type isolates [10]. Researches on HRSV evolution concentrate mainly on this domain in that the G protein is capable of stimulating neutralizing antibodies and is highly variable in antigenicity as well as genetics [18].

HRSVs have been classified as subgroups A and B (HRSV-A and HRSV-B) by both antigenic [19,20] and genetic analyses [10,21]. Up to now, 11 HRSV-A genotypes, GA1-GA5 [22], GA6-GA7 [23], SAA1 [24], NA1-NA2 [25], and ON1 [13], and 20 HRSV-B genotypes, GB1-GB4 [22], BA1-BA6 [26], BA7-BA10 [27], SAB1-SAB3 [24], SAB4 [28], URU1 and URU2 [29], have been identified based on sequence analyses of HVR2. The frequent appearance of new genotypes may facilitate HRSV to evade the existing herd immunity produced by previous infections of other genotypes, which might further influence disease severity [23] and cause larger outbreaks [25] and repeated infections [30].

HRSV has been a good model for the study of the relationship between epidemic progress and pathogen evolution [31]. The 60 nucleotide duplication of BA genotype in HRSV-B has been used as a natural tag to track the global transmission of HRSV [32]. Recently, a new HRSV-A genotype named ON1 with a 72 nucleotide duplication within HVR2 was reported in Canada [13]. Furthermore, surveillance on outbreaks of infections and molecular analysis of viruses is needed to investigate whether ON1 genotype is able to cause larger outbreaks and become the predominate genotype worldwide.

**Table 1:** Primers used in this study.

**PCR**	**Primer**	**Positions**	**Gene**	**Sequence^c^ (5'–3'**)	**Polarity**	**Reference**
**Subgroup-specific PCR**	GF	4677-4692^a^, 46-61^b^	G	CTAGAAARGACCTGGG	+	this study
	GAR	4928-4945^a^	G	AAGAAGCTGATTCCAAGC	-	this study
	GBR	382-398^b^	G	GTTGTATGGTGTGTTTC	-	this study
**G gene PCR**	S4298	4298-4319^a^	SH	TGGCCYTAYTTTACACTAATAC	+	this study
	F164	5764-5786^a^	F	GTTATGACACTGGTATACCAACC	-	21

^a^ Nucleotide positions are given according to strain A2 (GenBank accession number M11486).

^b^ Nucleotide positions are given according to strain CH18537 (GenBank accession number M17213).

^c^ Mismatches are underlined.

The study on the genetic variability of HRSV is necessary to better understand its molecular epidemiology, to predict future outbreaks and to develop an effective vaccine which has hitherto met with little success. Since there is a lack of information regarding this aspect for this most important virus in China, we carried out investigation on the genotype pattern and genetic variability of circulating HRSV strains isolated from infants and young children with ARI in Beijing in a period of 5 consecutive years.

## Materials and Methods

### Ethics Statement

This study was approved by the ethics committee of the Capital Institute of Pediatrics. No written informed consent was obtained because the samples used for this project were collected after routine laboratory testing, and the study was considered less than minimal risk to subjects by the aforementioned committee. The parents or guardians of the children underwent an oral consent process administered by both a clinician and a researcher. Both the clinician and the researcher signed their names in the registration forms to documented this process. The registration forms were obtained. The ethics committee of the Capital Institute of Pediatrics approved this consent procedure.

### Sample collection and HRSV antigen detection

Nasopharyngeal aspirates (NPA) were collected from patients who were hospitalized with ARI at the Affiliated Children's Hospital, Capital Institute of Pediatrics during the period from July 2007 to June 2012, and screened for the presence of antigens of 7 common respiratory viruses, including HRSV, influenza A and B, adenovirus and parainfluenza virus I-III, by immunofluorescent assay as a routine test. Indirect fluorescent assay (Chemicon, Temecula, CA, USA) and direct fluorescent assay (Diagnostic Hybrids, Athens, OH, USA) were used for specimens collected from July 2007 to June 2009 and from July 2009 to June 2012, respectively. Briefly, samples were homogenized with 5 ml Hanks solution shortly after collection and followed by centrifuge at 1237×g at room temperature for 5 minutes. The precipitates were used for antigen detection test by following the manufacturers’ instructions and the supernatants were then stored at -80°C for RNA extraction. Some of the supernatants were inoculated into Hep-2 cells for virus isolation before storing at -80°C.

### RNA extraction and reverse transcription (RT)

Total RNA was extracted directly from 150 μl of each randomly selected HRSV antigen-positive specimens or supernatant of cell-cultured isolates with Trizol agent (Invitrogen, Carlsbad, CA, USA) by following the manufacturer’s manual, and cDNA was synthesized by using random hexanucleotide primers (Invitrogen, Carlsbad, CA, USA) and moloney murine leukemia virus reverse transcriptase (Invitrogen, Carlsbad, CA, USA).

### Multiplex PCR and real time RT-PCR for subgroup-typing, PCR for full length attachment (G) gene

HRSVs were identified as subgroup A or B by using a multiplex PCR with primers GF, GAR and GBR designed according to the sequences of the reference strains in GenBank (Table 1). The PCR for subgroup typing was performed in a 25 μl final volume mixture containing 0.4 μM of each of subgroup A (GAR)- and subgroup B (GBR)-specific reverse primers and 0.4 μM of forward primer (GF), 3 μl of RT products, 0.2 mM deoxynucleoside triphosphates (dNTP), 1 U of DNA polymerase (TransGen Biotech, Beijing, China) and 2.5 μl PCR buffer (TransGen Biotech, Beijing, China) under the following thermocycling conditions: 94°C for 5 minutes, 35 cycles of denaturation at 94°C for 30 seconds, annealing at 49°C for 30 seconds, and extension at 72°C for 30 seconds, followed by a final extension at 72°C for 7 minutes. The amplified products of 269 bp for subgroup A and 353 bp for subgroup B were identified by electrophoresis in 2% agarose gels stained with ethidium bromide and visualized under UV light. Strain Long of prototype HRSV-A and strain CH18537 of prototype HRSV-B were successfully distinguished by this PCR procedure.

Antigen positive while multiplex PCR negative samples were retested by the real time RT-PCR, which primers and probes were designed according to the relative conserved N protein genes for subgroup-typing [33].

The full length attachment (G) genes of both HRSV-A and -B were amplified by PCR with primers S4298 and F164 (Table 1). The PCR was performed in a 25 μl reaction mixture using 0.4 μM of each of forward primer (S4298) and reverse primer (F164), 4 μl RT products, 0.2 mM dNTP, 1.5 mM MgCl_2_, 1 U of Platinum^®^ Taq DNA polymerase (Invitrogen, Carlsbad, CA, USA) and 2.5 μl PCR buffer (200 mM Tris-HCl (pH 8.4), 500 mM KCl) under the following thermocycling conditions: 94°C for 5 minutes, 45 cycles of denaturation at 94°C for 30 seconds, annealing at 49°C for 2 minutes, and extension at 72°C for 30 seconds, followed by a final extension at 72°C for 7 minutes. The products of 1489 bp were analyzed by electrophoresis in 2% agarose gels as aforementioned method. For some samples with weak signal in agarose gel, a 50 μl reaction mixture for PCR was performed in order to meet the quantity requirement for sequencing.

### Nucleotide sequencing

The PCR products from full length G genes were sequenced by Invitrogen using cycle sequencing in the forward and reverse direction with primers S4298 and F164.

**Figure 1:**
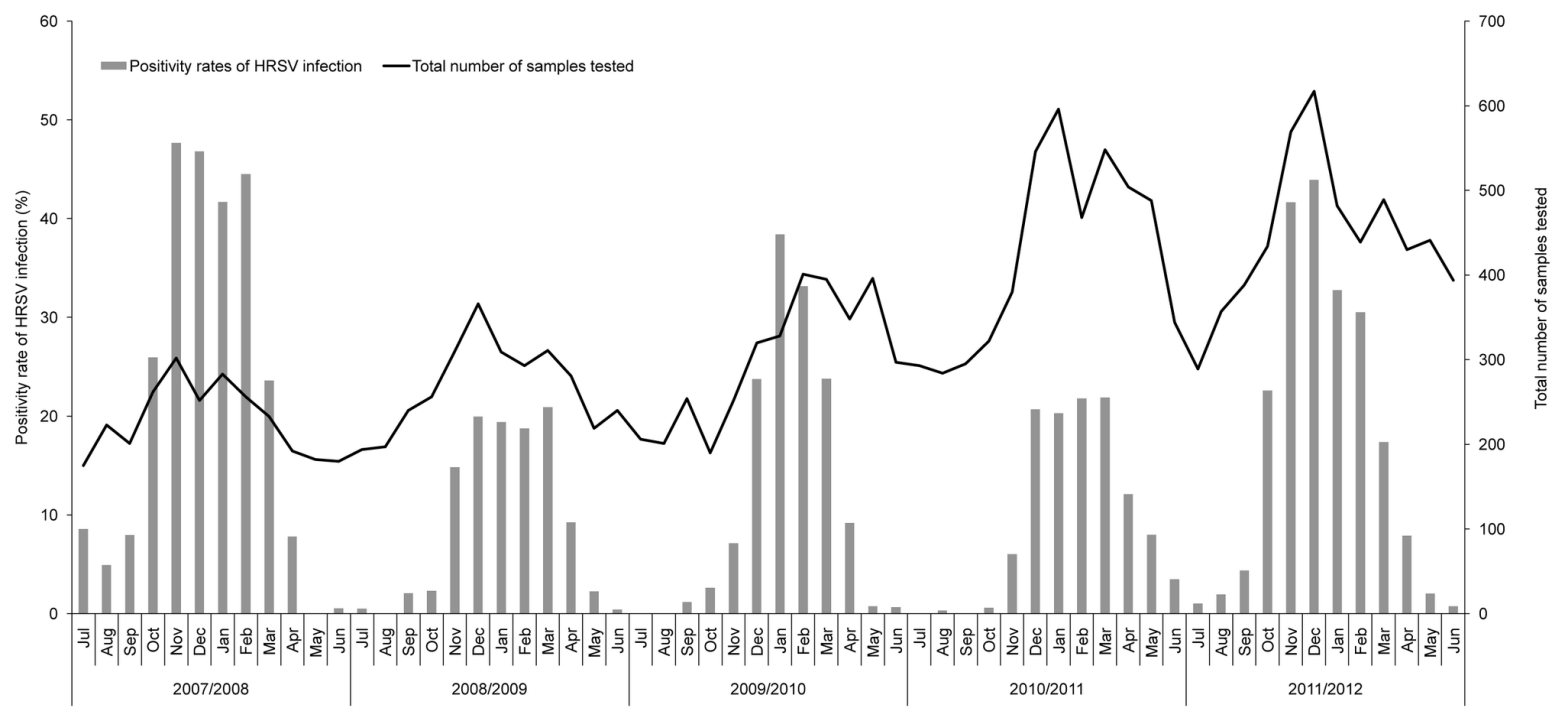
Monthly distributions of HRSV infection in Beijing during July 2007 to June 2012.

### Sequence analysis

Chromatogram files were inspected using Chromas Lite 2.1 (Thehnelysium, South Brisbane, Australia). Sequence assembly was performed with DNAStar 5.01 (DNASTAR, Wisconsin, USA). Identical sequences were identified with DAMBE software, version 5.3.10 [34].

### Phylogenetic analysis

Nucleotide sequences of G genes of HRSV with determined genotypes were obtained from GenBank for reference (Tables S1 and S2). Alignment of sequences from our study with reference sequences was performed on the ClustalW program within MEGA5 software [35]. Nucleotide sequences of HVR2 were aligned on the basis of the aligned predicted protein sequences. The overall mean nucleotide pairwise Jukes-Cantor distances of HRSV-A and -B alignment were 0.05 and 0.06, respectively. Phylogenetic trees of the HVR2 were constructed respectively for HRSV-A and -B alignment by the neighbor-joining method within MEGA5, using nucleotide p-distance for substitution model, complete deletion for gap or missing data treatment and 1000 replicates of bootstrap probabilities for evaluation of confidence estimates. The trees were unrooted.

### Glycosylation analysis

Potential N-glycosylation sites were predicted by sequence context of Asn-Xaa-Thr/Ser, where Xaa was any amino acid but proline.

### Statistical analysis

Significant differences in positivity rates of HRSV between males and females and between two HRSV seasons were tested with Pearson Chi-Square test using SPSS13.0 software. P-value of <0.05 was considered to be statistically significant.

### Nucleotide sequence accession numbers in GenBank

Nucleotide sequences of complete G genes identified in this study have been deposited in the GenBank under accession numbers KC297233 to KC297493 and KC559440.

**Figure 2:**
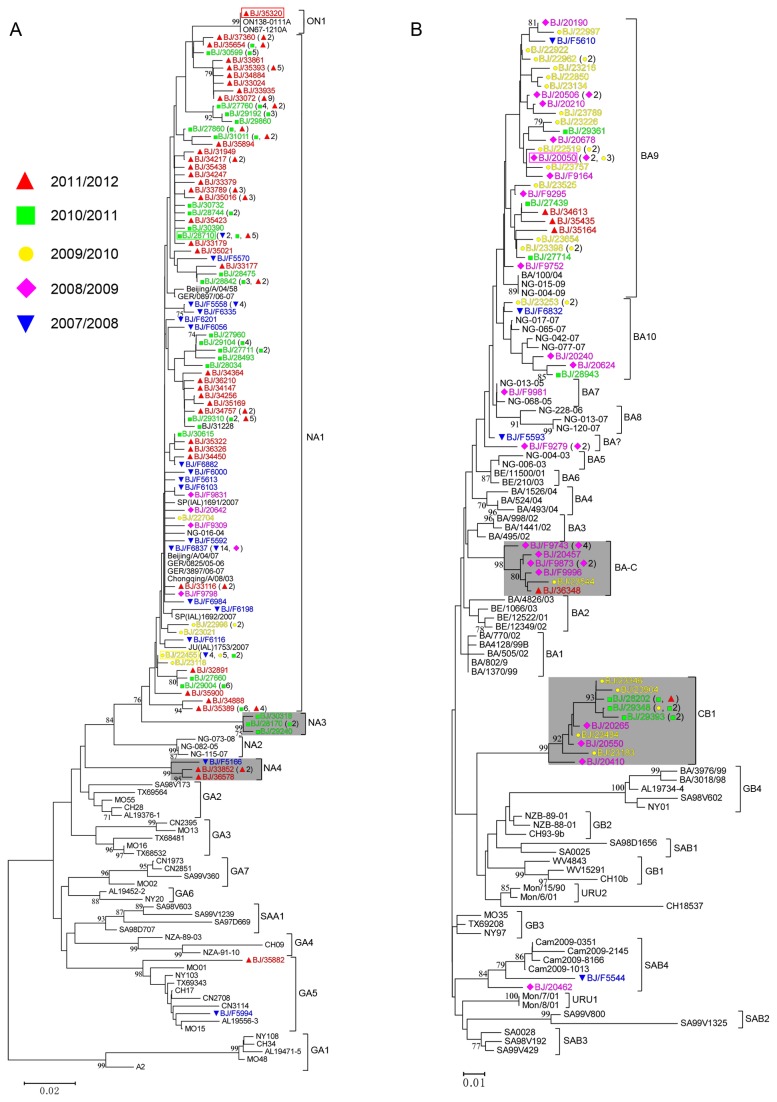
Unrooted phylogenetic trees of unique sequences of HVR2s of Beijing HRSV-A (A) and HRSV-B (B) strains and reference sequences of identified genotypes. Trees were constructed using the neighbor-joining method with 1,000 bootstrap replicates by Mega5 software. Only bootstrap values of >70% are shown at the branch nodes. The tree is drawn to scale, with branch lengths in the same units as those of the evolutionary distances used to infer the phylogenetic tree. The evolutionary distances were computed using the p-distance method and are in the units of the number of base differences per site. Beijing strains are colored and indicated by “BJ/”, followed by their isolation numbers. Samples collected from different epidemic seasons are indicated by colored strain names and colored symbols as follows: 2007/2008, ▼; 2008/2009, ◆; 2009/2010, ●; 2010/2011, ■; 2011/2012, ▲. Strain names mentioned in the text are labeled with rectangles to facilitate visualization. New genotype clades are indicated by gray shading The numbers of identical sequences of HVR2 are indicated in parentheses. The genotype assignment is indicated at the right by square brackets. “BA?” refers to HRSV-B strains with a 60 nucleotide duplication that are not assigned to any cluster.

## Results

### HRSV distribution

A total of 19,942 NPAs were collected from July 2007 to June 2012, including 7,397 (37.1%) from girls and 12,513 (62.8%) from boys. The gender information for 32 samples was missing. Out of the total NPA samples, 3,160/19,942 (15.8%) were positive for HRSV by antigen detection assay, including 1,079 from girls and 2,074 from boys. The positivity rate of HRSV in boys was significantly higher than that in girls (χ^2^=13.8, P=0.00).

Samples collected and antigen-positive rate of HRSV in each HRSV season are shown in Table 2. The overall positivity rates of HRSV infection in 2007/2008 and 2011/2012 were significantly higher than each of the other three HRSV seasons. A stronger HRSV activity with early detection and early annual peak of the virus in 2007/2008 season was followed by a weaker 2008/2009 season. This phenomenon has also been reported that a severe and early HRSV activity season is always followed by a weaker late season [36].

**Table 2:** Samples collection and HRSV subgroup A and B distributions in Beijing during July 2007 to June 2012.

**Epidemic season**	**Total No. of samples**	**No. (%**)**^a^ of antigen-positive samples**	**No. (%**)**^b^ of randomly selected samples**	**No. (%**)**^c^ of HRSV-A**	**No. (%**)**^c^ of HRSV-B**	**No. (%**)**^c^ of HRSV with undetected subgroup**	**No. of complete G gene sequences for HRSV-A**	**No. of complete G gene sequences for HRSV-B**
**2007/2008**	2741	675 (24.6^d^)	101 (15.0)	92 (91.1)	8 (7.9)	1 (1.0)	39	4
**2008/2009**	3216	343 (10.7)	102 (29.7)	12 (11.8)	87 (85.3)	3 (2.9)	5	27
**2009/2010**	3588	492 (13.7)	144 (29.3)	16 (11.1)	121 (84.0)	7 (4.9)	10	27
**2010/2011**	5068	594 (11.7)	180 (30.3)	162 (90.0)	12 (6.7)	6 (3.3)	57	9
**2011/2012**	5329	1056 (19.8^d^)	196 (18.6)	180 (91.8)	11 (5.6)	5 (2.6)	79	5
**all seasons**	19942	3160 (15.8)	723 (22.9)	462 (63.9)	239 (33.1)	22 (3.0)	190	72

a Percentages apply to the total number of samples.

b Percentages apply to the number of antigen-positive samples.

c Percentages apply to the number of randomly selected samples.

d Infection incidences in 2007/2008 and 2011/2012 epidemic seasons are significantly higher than each of other three epidemic seasons, 2008/2009, 2009/2010 and 2011/1012, by Pearson’s chi-square statistic.

The distribution of HRSV in each month is shown in Figure 1. HRSVs were detected from NPAs collected from every month except May and August in 2008, July and August in 2009, July and September in 2010 and July in 2012. HRSV annual epidemics occurred in winter, November to February, in all 5 investigated years. This clear cut seasonality is common in other northern hemisphere countries [25,37-39]. 

The ages from 16/3,160 HRSV positive patients were unknown. The minimum, lower quartile, median, upper quartile and maximum of ages of HRSV infected patients were 2 days, 2 months, 5 months, 16 months and 194 months, respectively.

### HRSV subgroup distribution

Of the 3,160 HRSV antigen-positive NPA specimens, 34 were excluded for further analyses, because these samples were also antigen-positive for other respiratory viruses. A total of 723 samples, including 687 random NPAs and 36 cell-cultured strains isolated from patients who were not overlapped with those 687, were selected for subgroup typing. Among them, 436 and 197 were determined as subgroup A and B by subgroup-specific multiplex PCR, respectively. Twenty-seven and 41 were further determined as A and B by real time PT-PCR, respectively, because they were not able to be typed by multiplex PCR. Twenty-two samples (3.0%, 22/723) were negative for both subgroup-specific multiplex PCR and real time RT-PCR (Table 2). These 22 samples were also negative for full length G gene PCR amplification (data not shown), perhaps because of a non-specific inhibitor of enzyme activity in these clinical specimens. Taken together, 65.9% (462/701) and 34.1% (239/701) of the HRSV were identified as group A and B, respectively. HRSV-A and HRSV-B were co-circulating during these 5 HRSV seasons (Table 2). Subgroup A were predominant during 3 seasons, 2007/2008, 2010/2011 and 2011/2012, while subgroup B were predominant during 2008/2009 to 2009/2010.

### Sequence alignments and phylogenetic analysis

Complete sequences of G genes from 190 HRSV-A and 72 HRSV-B were obtained and aligned with representative sequences of identified reference genotypes (Table 2). The phylogenetic trees and strains with identical nucleotide sequences of HVR2 are shown in Figure 2. According to the criteria for a novel genotype proposed by Venter - “sequences clustered together with bootstrap value of 70-100% and with a p-distance of less than 0.07 to all other members in the same phylogenetic cluster” [24], 4 new genotypes, NA3 and NA4 for HRSV-A, and CB1 and BA-C for HRSV-B were identified in this study (Figure 2, gray shading). The bootstrap values of the clusters NA3, NA4, BA-C and CB1 were ≥98%. Furthermore, the maximum p-distances of clusters NA3, NA4, BA-C and CB1 were 0.01, 0.02, 0.02 and 0.04, respectively. As shown in Figure 2, most of the identical HVR2 sequences were from strains isolated in the same HRSV season. However, there were identical sequences isolated from different seasons (e.g. strain BJ/28710 (Figure 2A, green rectangle) and strain BJ/22455 (Figure 2A, yellow rectangle) of genotype NA1, strain BJ/20050 (Figure 2B, magenta rectangle) of genotype BA9). In addition, the HVR2 nucleotide sequence of strain BJ/35320 (Figure 2A, red rectangle), containing a 72 nucleotide duplication, was identical to that of prototype ON1 strain ON67-1210A.

### Genotype distribution

Genotype distribution of HRSV in each HRSV season is shown in Table 3. Phylogenetic analysis revealed that 179/190 (94.2%) Beijing HRSV-A strains were classified as NA1 genotype, which was the dominant HRSV-A genotype during the five years. Genotype NA3 was only detected in 2010/2011 season. Genotype NA4 was detected in 2007/2008 and 2011/2012 seasons. One Beijing ON1 strain (BJ/35320) was found from one of the NPAs collected in February, 2012. Most (56/72) of Beijing HRSV-B strains belonged to BA genotype, of which 36 (50.0%) and 10 (13.9%) strains clustered in BA9 and BA-C, while 2 and 14 Beijing HRSV-B strains clustered into SAB4 and new genotype CB1, respectively. The CB1 genotype was detected during four HRSV seasons from 2008/2009 to 2011/2012 (also shown in Figure 2B, gray shading of CB1).

**Table 3:** HRSV genotype distributions in Beijing during July 2007 to June 2012.

	**NO. (%**)**^a^ of HRSV-A with the following genotypes**						**NO. (%**)**^a^ of HRSV-B with the following genotypes**							
**Epidemic season**	**Total^b^**	**NA1**	**NA3**	**NA4**	**ON1**	**GA5**	**Total^c^**	**BA7**	**BA9**	**BA10**	**BA-C**	**BA? ^d^**	**SAB4**	**CB1**
**2007/2008**	39	37 (94.9)	0	1 (2.6)	0	1 (2.6)	4	0	1 (25)	1 (25)	0	1 (25)	1 (25)	0
**2008/2009**	5	5 (100)	0	0	0	0	27	1 (3.7)	10 (37.0)	2 (7.4)	8 (29.6)	2 (7.4)	1 (3.7)	3 (11.1)
**2009/2010**	10	10 (100)	0	0	0	0	27	0	19 (70.4)	2 (7.4)	1 (3.7)	0	0	5 (18.5)
**2010/2011**	57	53 (93.0)	4 (7.0)	0	0	0	9	0	3 (33.3)	1 (11.1)	0	0	0	5 (55.6)
**2011/2012**	79	74 (93.7)	0	3 (3.8)	1 (1.3)	1 (1.3)	5	0	3 (60.0)	0	1 (20.0)	0	0	1 (20.0)
**all seasons**	190	179 (94.2)	4 (2.1)	4 (2.1)	1 (0.5)	2 (1.1)	72	1 (1.4)	36 (50.0)	6 (8.3)	10 (13.9)	3 (4.2)	2 (2.8)	14 (19.4)

a Percentages apply separately to the number of HRSV-A and - B in each epidemic season.

b The number of the G gene sequences of HRSV-A.

c The number of the G gene sequences of HRSV-B.

d “BA?” represents HRSV-B strains with the 60 nucleotide duplication not clustering into any other identified BA genotypes.

### Amino acid analysis

**Figure 3:**
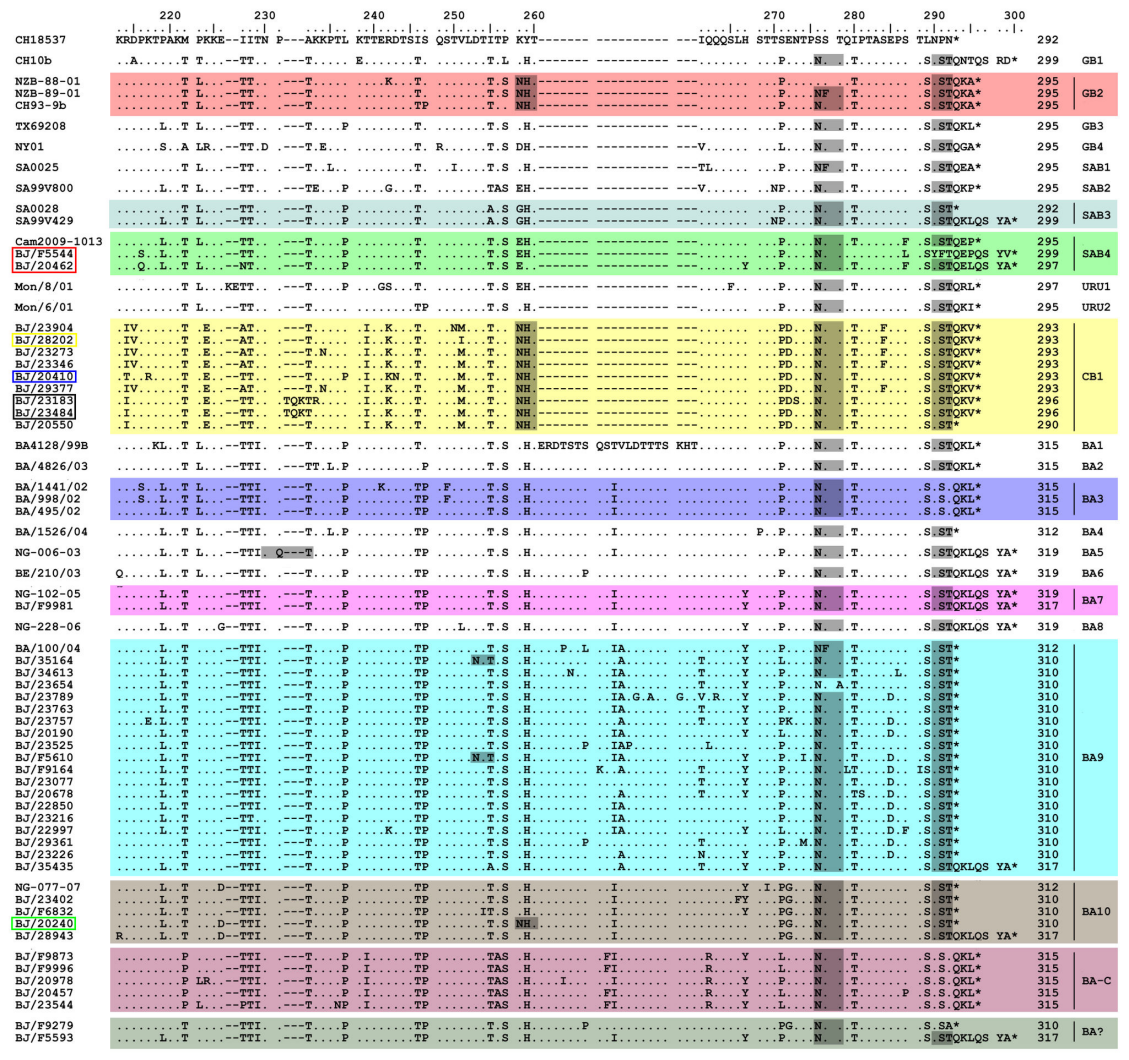
Alignments for deduced amino acids of HVR2s from Beijing HRSV-B strains and representative reference strains of identified HRSV-B genotypes. Amino acid numbering is shown relative to prototype CH18537 (GenBank accession number M17213). Only Beijing strains with unique HVR2 sequences are included in the alignments. Identical residuals, stop codons and gaps are indicated by dots, asterisks and dashes, respectively. Genotype names, followed by the length of G proteins, are shown at the end of the sequences. The G protein length of Beijing strains are deduced from the full length of G protein, while the G protein length of reference strains are given according to prototype of A2. Potential N-glycosylation sites (NXT, where X is not proline) are indicated by gray shading. Different background colors are used to facilitate visualization of different genotypes.

**Figure 4:**
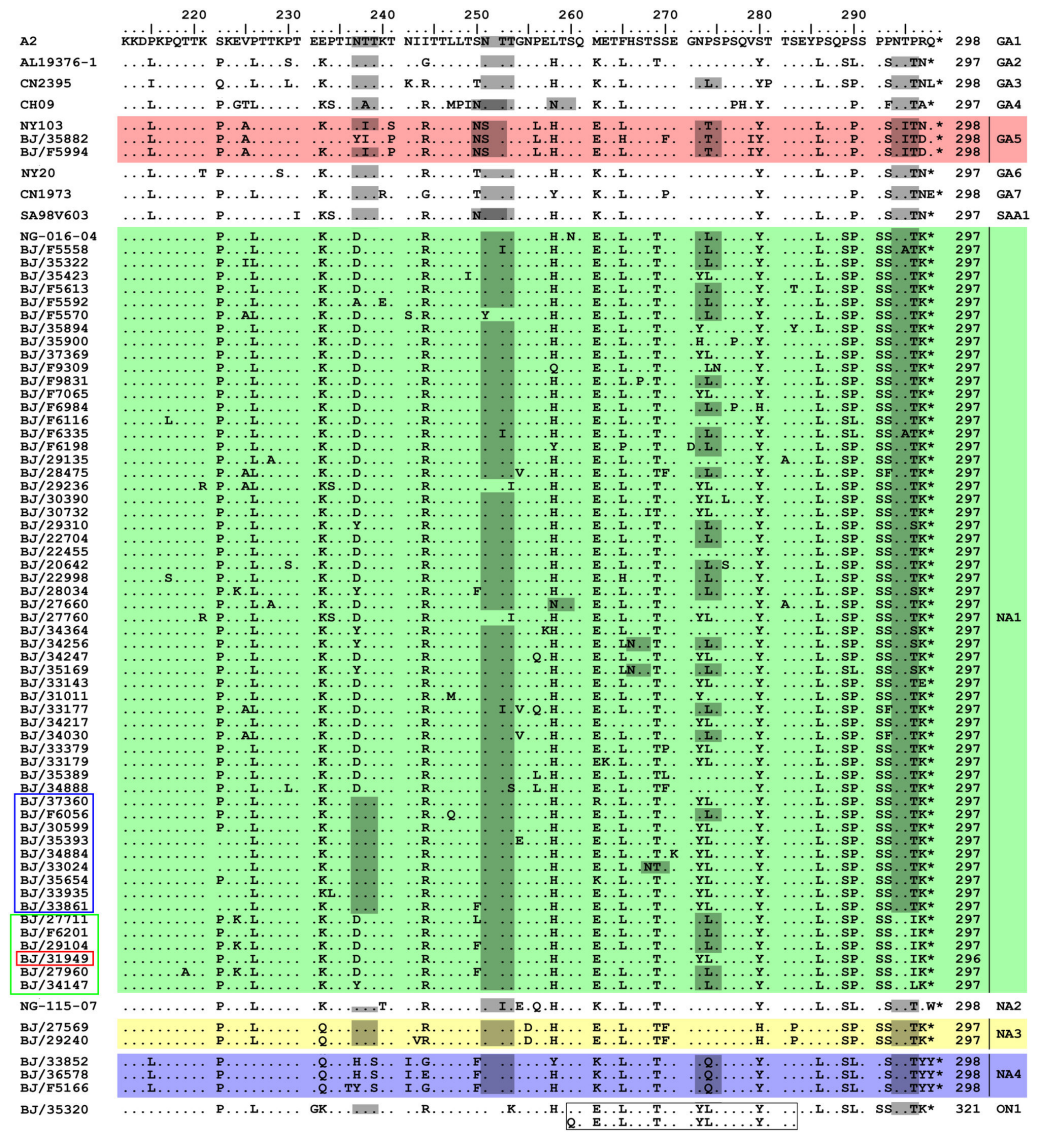
Alignments for deduced amino acid of HVR2 from Beijing HRSV-A strains and representative reference strains of identified HRSV-A genotypes. Amino acid numbering is shown relative to prototype A2 (GenBank accession number M11486). Only Beijing strains with unique HVR2 sequences are included in the alignments. Identical residuals and stop codons are indicated by dots and asterisks, respectively. Genotype names, followed by the length of G proteins, are shown at the end of the sequences. The G protein length of Beijing strains are deduced from the full length of G protein, while the G protein length of reference strains are given according to prototype of A2. Potential N-glycosylation sites (NXT, where X is not proline) are indicated by gray shading. Different background colors are used to facilitate visualization of different genotypes. The 24 amino acid duplication in the genotype ON1 are shown in the rectangle.

The lengths of all G proteins from Beijing HRSV were deduced based on the complete coding sequences of G genes. The predicted lengths of G proteins from HRSV-B were between 290 and 319 amino acids (Figure 3). It should be noted that a 9 consecutive nucleotide insertion (ACACAAAAA, coding for Thr(T)-Gln(Q)-Lys(K)) occurred next to residue 693 of the G gene (relating to HRSV-B prototype strain CH18537) in the HVR2 in 3 of CB1 strains, BJ/23484, BJ/23183 (Figure 3, black rectangle) and BJ/20265, leading to their G proteins lengthened by 3 amino acids. In addition, although both BJ/F5544 and BJ/20462 (Figure 3, red rectangle) strains clustered in SAB4 genotype, the G protein length of BJ/F5544 was 2 amino acids longer than that of BJ/20462 due to a 6 consecutive nucleotide in-fame deletion in the highly conserved region of G protein of BJ20462 (CCAAAA, residue 475-480 compared to prototype strain CH18537, coding for Pro(P)-Lys(K), data not shown). This deletion was first reported by Zlateva [12] and also seen in genotypes BA7, BA9, BA10 and CB1 of Beijing isolates. The G protein length for HRSV-A was either 297 or 298 amino acids, with the exception of ON1 genotype, which was 321 amino acids due to a 72 nucleotide duplication in HVR2 (Figure 4). However, the G protein length of the Beijing HRSV-A strain BJ/31949 (Figure 4, red rectangle) was 296 amino acids due to a 3 consecutive nucleotide deletion in the highly conserved region of G gene (AAC, residue 571-573 compared to HRSV-A prototype strain A2, data not shown).

Several genotype-specific substitutions were found in the novel genotypes identified here. Substitutions N255D and S280H were restricted in the HRSV genotype NA3 (Figure 4). The genotype NA4 was characterized by T293S, N242I and P274Q (Figure 4). Genotype BA-C could be defined by M222P, T256A and Q262R. Genotype CB1 could be defined by K224E (Figure 3). Additionally, substitutions R214I and V251M were restricted in strains of CB1 genotype, except for strain BJ/20410 and BJ/28202 (Figure 3, blue and yellow rectangle, respectively). Furthermore, substitution T239I was found only in new genotypes CB1 and BA-C (Figure 3).

### Glycosylation analysis

Five major putative N-glycosylation sites, N237, N250, N251, N273 and N294, were identified in HVR2 of HRSV-A (Figure 4, gray shading). Although the first site N237 was conserved in most of genotypes of HRSV-A, it was missing in genotype NA4 and most members of the genotype NA1. However, this site was present in 9 Beijing NA1 strains (Figure 4, blue rectangle). The second site N250 was specific for genotype GA5 and SAA1. All HRSV-A genotypes contained the fifth site N294, except prototype A2 and 6 Beijing NA1 strains (Figure 4, green rectangle). As for HRSV-B (Figure 3), it was interesting to find out that the substitution K258N resulted in an N-glycosylation site in all CB1 strains, which also existed in GB2 genotype and one strain BJ/20240 (Figure 3, green rectangle), but not in any other HRSV-B genotypes. In addition, all BA-C strains lost the N-glycosylation site of N290, which was the same as the prototype CH18537 and genotype BA3.

## Discussion

Combining two other Beijing studies [40,41] with this study, a shifting mixed pattern of subgroup dominance was observed in Beijing during 12 HRSV seasons, namely, BAAABAAABBAA, where A and B represent HRSV-A and -B dominance, respectively, in the order of years from 2000 to 2012. Most studies have revealed that viruses of both subgroups co-circulated during each HRSV season, but the shifting pattern of predominant subgroup varied [28,39,42,43]. This reflects the localized character of herd immunity and the ability of HRSV to evade this herd immunity, challenging the development of a globally effective HRSV vaccine.

Although NA3 and NA4 were considered as emerging HRSV-A genotypes identified here, they were detected from a few specimens collected in certain years (Table 3); NA1 was the prevailing HRSV-A genotype during these five years. Several studies have reported an annual shift of the dominant genotype [22,44,45]. However, our study revealed that NA1 genotype predominated during two consecutive HRSV seasons. Previous studies also found out that NA1 genotype predominated during two successive epidemics both in Japan [25] and Cambodia [28], suggesting that NA1 strains was capable of evading the host immune response. Interestingly, all NA1 strains isolated from Japan lost the first N-glycosylation site due to substitution N237D [25], while this N-glycosylation site emerged in 9 Beijing NA1 strains (Figure 4, blue rectangle). Furthermore, disappearance of the fifth N-glycosylation site was also found from 6 Beijing NA1 strains (Figure 4, green rectangle). Changes of glycosylation in the G protein may help the virus to alter antigenic characteristics [16,46], leading to an evolutionary advantage.

First identified in Buenos Aires [47], the genotype BA with a 60 nucleotide duplication within HVR2 has become the predominant HRSV-B worldwide [25,26,28,39,48,49], including northwest China [50] and southwest China [38]. The BA genotype in China was first detected in Beijing by our team [40]. In this present study, we found that BA was the predominant HRSV-B genotype in Beijing, with BA9 being the most frequently detected branch. The prevalence of BA9 has been recently reported in Japan [27], Korea [51] and Philippines [52]. However, the new BA genotype, BA-C, and genotype CB1 without the 60 nucleotide duplication were detected in at least 3 HRSV seasons (Table 3 and Figure 2B, gray shading). The herd immunity induced by the widespread infection of BA9 in the current population may drive the emergence of the new genotypes BA-C and CB1. Interestingly, loss of N-glycosylation sites in BA-C genotype and addition of N-glycosylation sites in CB1 genotype resulted in the same glycosylation states as previous genotypes BA3 and GB2, respectively (Figure 3). It is of note that K258 in HRSV-B has been identified as a positively selective site [12]. The positive selection pressure may drive the emergence of substitution K258N, and, thus, lead to an N-glycosylation site in new CB1 genotype. This reversal glycosylation pattern may give the virus the ability to survive in the current state of herd immunity. In fact, reversible evolution has been suggested as a strategy for the virus to escape the changing immune status of the human population [53]. Subsequent investigations are needed to evaluate whether changes of putative glycosylation pattern are associated with antigenic variability in these new HRSV genotypes.

Here, we reported the earliest found ON1 strain so far in China, BJ/35320, which was isolated from one of the specimens collected in February, 2012. First detected in a sample collected on December 29^th^, 2010 [13], the ON1 genotype has been found in countries of almost all continents during the following two years, including Japan (GenBank accession number AB700370), South Korea [54], Germany (GenBank accession number JX912357), South Africa [55], Malaysia [56] and Italy (GenBank accession number JX988439). This reflects the rapid global transmission of genotype ON1. During the preparation of this manuscript, several ON1 strains had been detected from clinical samples collected in the winter of 2012. It is of great interest to know, as found in genotype BA discussed above, whether ON1 would become the predominant genotype for HRSV-A worldwide. Moreover, much more attention should be paid to the genetic variation of this genotype, although no mutation in the HVR2 nucleotide sequence was found in ON1 strains isolated in South Africa [55], Malaysia [56] and here.

Information on the clinical diagnoses of 199 children infected with identified genotypes was obtained (Table S3). Most (94.5%, 188/199) of HRSV infected children were diagnosed with bronchiolitis or pneumonia, as reported by Oliverira et al [57] and Zhang et al [50]. Because only a few cases infected with novel genotypes have been found, the role of these genotypes in determining disease severity needs further investigation.

The G protein length polymorphism from HRSV-B was much higher than that of HRSV-A, in which change of the length arose overwhelmingly from amino acid substitutions, with exception of genotype ON1 and strain BJ/31949 (Figure 4, red rectangle). To our knowledge, the three consecutive nucleotide deletion in the highly conserved region of G gene in strain BJ/31949 is first reported here. Reasons for the variability among the G protein lengths of HRSV-B include amino acid substitutions, insertions and changes of stop codon usage (Figure 3). We describe here for the first time that a 9 consecutive nucleotide insertion in HVR2 of 3 Beijing strains, BJ/20265, BJ/23484 (Figure 3, black rectangle) and BJ/23183 (Figure 3, black rectangle). The association of changes in G protein length with antigenic variation has been reported [58,59]. The antibody response induced by HRSV-A might elicit more extensive cross-protection against HRSV-B than vice versa [60], so HRSV-B might need more extensive genetic variation (e.g. changes of G protein length) to evade herd immunity.

Most strains with identical HVR2 sequences were found circulating in the same HRSV season (Figure 2), suggesting a slight immunological advantage by evading existing immunity. However, strains with identical sequences could also be found in different HRSV seasons and from distant cities in China by using the BLAST. For example, the HVR2 nucleotide sequences of strain BJ/23346 of genotype CB1 isolated in 2010 was identical to strains LZY148, LZY115, LZY113, LZY83 and LZY16 isolated from Lanzhou city, northwestern China, in 2008 [50]. Strain BJ/F9873 of genotype BA-C had the same nucleotide sequences of HVR2 with strain B012703 (GenBank accession number JF13440) isolated from Chengdu city, southwestern China. These findings may reflect concurrent circulation of emerging strains facilitated by mass migration movements in China.

However, strains with identical sequences of HVR2 were sometimes classified as different genotypes in different studies. For examples, strain BJ/23346 identified as new genotype CB1 in this study was identical to five strains from Lanzhou mentioned above, which were classified as genotype GB2 [50]. In addition, strains ChongqingB/09/23 and ChongqingB/06/02, first classified as genotype GB3 [38], were reclassified as genotype SAB4 [28]. This may due to the use of different software, nucleotide substitution models and various algorithms in different studies. Thus, we suggest a general criterion for HRSV genotyping to make comparison studies more effective and accurate.

The limitation of this study is that all the samples were collected from hospitalized patients in only one hospital in China, but such data was suggested to be useful for extrapolation of epidemics as a whole and comparison study [31].

In summary, although four novel genotypes were found in Beijing, namely NA3, NA4, BA-C and CB1, they were not predominant during 2007 to 2012. However, changes of codon states for the G protein back to previous ones in these new genotypes as well as in the prevailing genotypes might allow their survival in the current state of herd immunity. Ongoing and long-term molecular epidemiological surveillance is necessary to better understand this important virus.

## Supporting Information

Table S1 **Reference sequences of identified genotypes of HRSV-A downloaded from GenBank.**
(XLS)

Table S2 **Reference sequences of identified genotypes of HRSV-B downloaded from GenBank.**
(XLS)

Table S3 **Information for diagnoses of 199 children infected with identified genotypes.** ^a^ “BA?” represents HRSV-B strains with 60-nucleotide duplication not clustering into any other identified BA genotypes. URTI is short for Upper Respiratory Tract Infection.
(XLS)
